# Challenges of providing biochemistry results in a patient with Evans syndrome

**DOI:** 10.11613/BM.2024.011001

**Published:** 2023-12-15

**Authors:** Natividad Rico Ríos, Alison Bransfield, Caroline M Joyce, Mary R Cahill, Michelle O’Shaughnessy, Seán J. Costelloe

**Affiliations:** 1 Department of Clinical Biochemistry, Cork University Hospital, Cork, Republic of Ireland; 2 Department of Clinical Haematology, Cork University Hospital, Cork, Republic of Ireland; 3 Department of Nephrology, University Hospital Galway, Galway, Republic of Ireland

**Keywords:** Evans syndrome, hemolysis, *in vivo* hemolysis, case report

## Abstract

A case report of *in vivo* hemolysis in a female patient with Evans syndrome is described. The patient was admitted with anemia and jaundice and, during her 26-day hospital admission, had 83 samples taken for biochemistry analyses. The laboratory hemolytic index (HI) was frequently elevated due to persistent complement-mediated *in vivo* hemolysis despite multiple lines of therapy. Initially, the release of many biochemical parameters was blocked *per* the manufacturer´s recommendations and reported as “sample hemolyzed”. The patient developed severe acute kidney injury, ultimately requiring dialysis. Automated and timely reporting of indicative creatinine and other biochemical results in the context of ongoing hemolysis, therefore, became essential to patient care. Following a review of literature from various sources, a laboratory algorithm was designed to ensure the timely release of numerical biochemical values, where possible, with appropriate interpretative comments appended. Biochemistry, hematology, and nephrology teams were in regular communication to ensure patient samples were rapidly identified, analyzed and validated according to the algorithm, informing timely, safe and appropriate patient care. Ultimately, the patient died due to multiple disease- and treatment-related complications. In conjunction with clinical users, laboratories should plan for situations, such as *in vivo* hemolysis, where significant unavoidable interferences in biochemistry methodologies may occur in an ongoing manner for certain patients. Reporting categorical or best-estimate biochemistry results in such cases can be safer for patients than failing to report any results. Interpretation of these results by clinical teams requires input from appropriately trained and qualified laboratory personnel.

## Highlights

A case report of *in vivo* hemolysis in patient with Evans syndrome is described

Hemolysis disrupts biochemistry analysis, yielding unreliable results

A laboratory designed algorithm ensures results with interpretative comments

Close communication between the laboratory and the clinical team is essential

## Introduction

Evans syndrome (ES) is a rare syndrome combining, either sequentially or concomitantly, autoimmune hemolytic anemia (AHA) with immune thrombocytopenia (ITP) or thrombocytopenic purpura and, in certain cases, autoimmune neutropenia (*1*). Evans syndrome can occur as a primary or secondary disease, the latter being associated with lymphoproliferative disorders, solid tumours, autoimmune and inflammatory diseases, viral infections or primary immunodeficiency. Evans syndrome typically manifests as a chronic disease with acute episodes and has a mortality rate between 10 and 20% due to relapse and severe anemia with or without thrombotic and infectious complications (*1*, *2*).

Although there is no standardised protocol, laboratory diagnostic assessments, including direct Coombs’ test (DCT) (positive), blood film for spherocytes and reticulocytes (increased), lactate dehydrogenase (LD) (increased), bilirubin (increased) and a low platelet count (often below 30 x10^9^/L) can support the diagnosis of ES (*3*, *4*). Following diagnosis, assessing the severity of hemolysis and response to treatment is critically dependent on biochemistry results.

Management of ES includes first-line corticosteroids and intravascular immunoglobulins to prevent bleeding episodes, second-line rituximab, and potential third-line immunosuppressant therapy or splenectomy (*5*). Any underlying condition, such as leukemia or lymphoma, also requires active and specific management.

Both *in vitro* and *in vivo* hemolysis can release cell-free hemoglobin (fHb) from red blood cells (RBCs) into the serum or plasma, interfering with the quantification of many routine biochemistry parameters. The degree of interference depends on the grade of hemolysis, and this property is often quantified as a hemolysis index (HI), corresponding to a fHb concentration value or range. Unlike *in vitro* hemolysis, where specific analytes can appear in falsely high concentrations when *in vivo* hemolysis occurs, the contents of the erythrocyte can circulate throughout the vascular space, allowing some released components to equilibrate with the interstitial fluid. *In vivo* hemolysis does not, therefore, always result in spuriously high analyte concentration; however, the fHb concentration can be high enough in serum or plasma to cause methodological interference.

Although modern automated analyzers can detect hemolysis, they cannot distinguish the etiology. It is essential to have processes in place for distinguishing different types of hemolysis interference, as these will aid with the clinical interpretation and release of results. Typical signs of *in vivo* hemolysis include the reduction of serum or plasma haptoglobin concentration as fHb is bound and removed from the circulation; the elevation of indirect bilirubin as fHb released from RBCs is metabolised; and an elevated reticulocyte count, an indicator of the bone marrow compensatory response.

Here, we describe the case of a 40-year-old woman presenting to the emergency department (ED) with jaundice, headache and palpitations. Her medical history included *a prior* diagnosis of ES with AHA, ITP and splenectomy in childhood. The patient had good health for 11 years before presentation when she required steroids and intravenous immunotherapy to control intermittent relapses. Herein, we describe the challenges of reporting clinically useful laboratory data for a patient with an extended hospital stay and persistent *in vivo* hemolysis.

## Laboratory analyses

The patient had 83 blood samples (for 1204 tests) sent to the biochemistry laboratory during her 26-day hospital episode. The HI was consistently elevated (HI ≥ 4), with 94% indicating hemolysis during the hospital episode. Electronic rules on the laboratory middleware (Remisol Advance, Beckman Coulter, Brea, USA) and laboratory information management systems (LIMS, iLab version 6) initially blocked the release of many parameters *per* manufacturer recommendations. Hemolysis index was measured on a Beckman Coulter AU5800 analyzer (Beckman Coulter, Brea, USA) which uses spectrophotometric method and provides an ordinal scale to grade hemolysis from “0” to “+++++”. Biochemistry results were reported as “Sample hemolyzed” by default when the value of the HI for a specific analyte was over the cut-off established by the manufacturer (Table 1).

**Table 1:** Beckman Coulter (AU5800 analyzer) Hemolysis Index cut-offs and analytes affected

**HI**	**Instrument flag**	**Concentration range of fHb (g/L)**	**Analytes affected**
1+	+	0.5-0.99	AST Direct Bilirubin Iron LD Potassium* Ammonia Total Bilirubin
2+	++	1-1.9	CK Magnesium ACE
3+	+++	2-2.9	Sodium Chloride Total Protein
4+	++++	3-5	Cholesterol Phosphate* Paracetamol Troponin
5+	+++++	> 5	All Remaining analytes
The points on the hemolysis index (HI) ordinal scale (flags) used on the Beckman AU 5800 analyzer (Beckman Coulter, Brea, USA) and the serum or plasma hemoglobin concentration to which the index corresponds are given. This information is obtained from the manufacturers pack insert of each analyte. *Potassium HI is lower than phosphate HI, this may protect from interference caused by spuriously hyperkalemia but not hyperphosphatemia. HI - hemolysis index. fHb - cell-free hemoglobin. AST - aspartate aminotransferase. LD - lactate dehydrogenase. CK - creatine kinase. ACE - angiotensin-converting enzyme.			

## Clinical course, interventions and further investigations

Initial complete blood count (CBC) results showed a total Hb of 71 g/L (reference interval (RI): 117-155 g/L), with a total bilirubin of 102 µmol/L (RI: 2-20 µmol/L) and lactate dehydrogenase (LD) of 2097 U/L (RI: 225-450 U/L). The patient was initially treated with intravenous prednisolone (1 mg/kg), later escalated to high-dose methylprednisolone on day three of her hospital episode in the setting of ongoing potentially life-threatening hemolysis and Rituximab (375 mg/m^2^) was added on day five. A pulmonary embolus, a common complication of uncontrolled hemolysis, was detected on day five. Coagulation results were unreliable due to the interference caused by the high hemolysis observed in this period.

For the first two days of hospital admission the majority of biochemistry results requested were able to be reported, with the exception of LD and total bilirubin due to these assays being more sensitive to interference at lower grades of hemolysis. However, from day two onwards, the HI was consistently elevated such that automatic reporting of many laboratory parameters was blocked, and an automatic “sample hemolyzed” comment was appended *per* historical laboratory practice at this centre. At this time, biochemistry laboratory personnel and the treating hematology team discussed the patient’s case, and a decision was made to release additional results to facilitate patient management as the patient’s clinical situation was rapidly deteriorating. The biochemistry consultant cautiously advised on likely result ranges and broad trends for those parameters affected by hemolysis. Verbal results and advice were recorded in the LIMS, and a direct line of communication was established between clinical and laboratory personnel.

**Figure 1:**
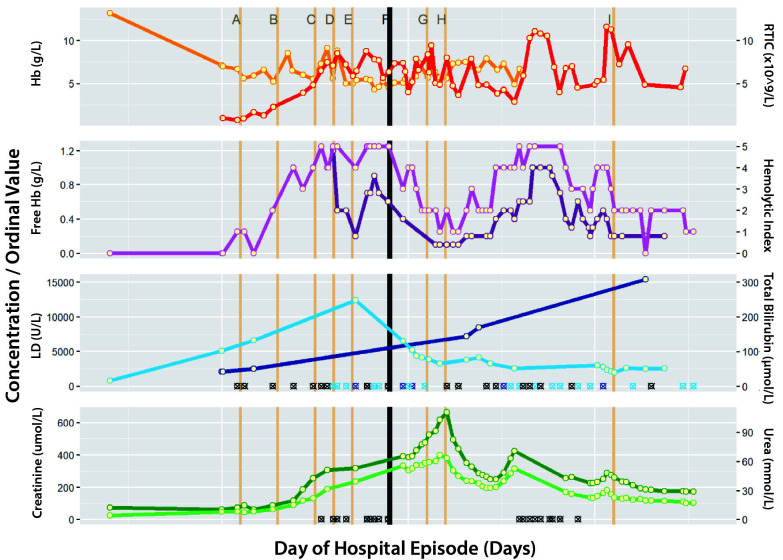
Biochemistry analytes trends during hospital stay (26 days). Y axis: concentration of analytes. X-axis: hospital stay in number of days. Certain analytes are graphed on different axes and these are colour-coded (Hb - mustard yellow, reticulocyte count - red, fHb - dark purple, HI - light purple, LD - dark blue, total bilirubin - light blue, creatinine - dark green, urea - light green). Each graph contains filled circles to indicate when measurements were made. Along the x axis, small crossed boxes points indicate where tests were requested but not reported (coloured with the same colour as the analyte series and coloured black when both analytes were requested at the same time point). Perpendicular yellow lines divide the graphs into periods that correspond to different scenarios that occurred (clinical management actions, laboratory algorithm implementation and patient clinical situation): A. Day 1. intravenous prednisolone (1 mg/kg). B. Day 3. High-dose methylprednisone. C. Pulmonary embolism identified. Rituximab (375 mg/m^2^) added to regimen. D. Respiratory compensation and oliguria. E. Bendamustine added. F. Algorithm introduced. G. Abdominal pain, partial vein thrombosis, large bowel iscahemia identified. H. Dialysis begins. I. Ischaemic bowel resection. Vertical black line indicates the day the algorithm was introduced. RtlC - reticulocyte count. Hb - hemoglobin. fHb - cell-free hemoglobin. HI - hemolysis index. LD - lactate dehydrogenase.

On day six, respiratory compromise and oliguria developed, and the patient’s overall clinical condition deteriorated further. Although severe acute kidney injury was suspected, the HI of “5+” automatically blocked the release of urea and creatinine values. Although results were not available to the clinical team at the time, it was subsequently suggested that that creatinine and urea concentrations had risen five-fold, reaching values of > 300 µmol/L and > 30 mmol/L, respectively, by day five, subsequently peaking at 663 µmol/L and 66 mmol/L, respectively, on days 12 and 11 respectively (Figure 1). A dialysis catheter was placed and hemodialysis was initiated on day 12.

On day seven, bendamustine was added to combination immunosuppressive therapy. Antibiotic therapy was also commenced due to clinical evidence of pneumonia upon computed tomography of the thorax.

Plasmapheresis with albumin was commenced on day ten.

Abdominal pain with portal vein thrombosis and large bowel ischemia developed on day 11. On this same day, following lengthy national and local discussions, eculizumab (dose of 900 mg) was procured and administered, after which there appeared to be a dramatic but temporary positive effect on the rate of intravascular hemolysis, reflected in the HI with the patient’s port wine-coloured urine becoming almost yellow and much more transparent. An increase in total Hb was seen between day 11 (51 g/L) and day 13 (70 g/L) following the administration of two units of red cell concentrates. This rise in total Hb was not sustained, and on day 14, the total Hb dropped to 57 g/L, with an increase in bilirubin (from 75 to 100 mmol/L) and LD (from 6000 to 7400 U/L), respectively). At this point, there was continuous combined therapy of rituximab, methylprednisolone, and red cell concentrates.

On day 21, a resection of the ischemic bowel was performed. Following complications of bowel ischemia, sepsis and thrombosis in conjunction with the underlying hemolysis, the patient died on day 26.

Throughout these events, regular communication between the biochemistry, hematology, and nephrology teams occurred, but despite these verbal communications, it was felt that a more automated and seamless procedure was needed to allow for the safe and prompt release of results. To this end, an algorithm was developed and implemented on day nine, as described.

## Solution

**Figure 2:**
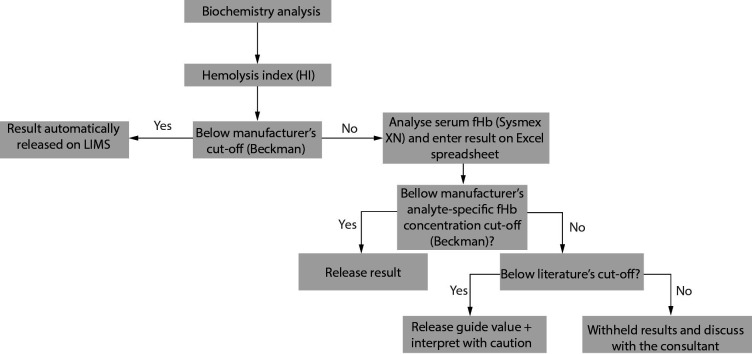
Laboratory algorithm for release of results for a patient with Evans syndrome. The procedure was implemented in Excel (Microsoft, Washington, USA) and it allowed the release of the greatest number of numeric and guide values for biochemical parameters. Every sample had a HI and an fHb analysis. If the HI was below the manufacturer´s cut-off for any specific parameter, the results were automatically released. If the HI was above the manufacturer´s cut-off, the fHb result was entered into the Excel spreadsheet and an automatic comment was obtained. When fHb concentration was below the manufacturer´s cut-off for a specific parameter, then the comment obtained was “release result”; when it was below the literature´s cut-off, the comment obtained was “release guide value and interpret with caution recommendation”; and when the fHb was above the manufacturer´s cut-off for a specific analyte and above literature´s cut-off, the comment obtained was “withheld the results and discuss with the consultant”. fHb - cell-free hemoglobin. HI - hemolysis index.

The biochemistry laboratory team developed an algorithm (Figure 2) in Microsoft Excel (Microsoft, Washington, USA) based on the manufacturer’s data, published cut-offs for fHb interference in a single study, and direct measurement of fHb (Table 2) (*6*). Following implementation on day nine of the hospital episode, this process was followed for all specimens received in the biochemistry laboratory for this patient. Serum fHb was quantified using a cyanide-free sodium lauryl sulphate (SLS) method (XN, Sysmex, Kobe, Japan). Total Hb analysis is part of the CBC profile, and the method is validated for whole blood samples. This method has a limit of detection (LOD) of 10 g/L, precision of measurement of 1.5% and measuring range linearity from 0 to 250 g/L. Despite the absence of a validated routine method for analysis of fHb in plasma or serum in the marketplace, the whole blood method principle routinely used for measuring total Hb was considered appropriate for measuring fHb in this urgent clinical scenario and the algorithm as described. As stated in Figure 1, there was a lower trend of values for fHb than for HI for the same samples. This difference on fHb estimation could be due to the different methology of measurement (SLS *vs* spectrophotometry) and sample type (whole blood *vs* serum).

**Table 2:** Analyte specific fHb cut-off (g/L) based on the manufacturer´s information and literature evidence

**Analyte**	**Manufacturer´s cut-off (fHb, g/L)**	**Literature cut-off (fHb, g/L)**
Sodium	5	7.9
Potassium	0.5	0.8
AST	0.25	0.25
LD	0.25	0.25
Cl	5	7.9
Creatinine	5	7.9
Urea	5	7.9
Total bilirubin	5	7.9
ALT	5	1.5
ALP	4.5	7.9
Bicarbonate	5	7.9
Phosphate	3.5	3.9
Calcium	5	7.9
Total Protein	5	7.9
Albumin	4.5	3.9
CRP	5	-
This table shows the fHb cut-offs for each analyte used to elaborate the algorithm. The first column states the cut-offs based on the manufacturer´s (AU5800, Beckman Coulter, Brea, USA) pack insert information, and the second column states cut-offs based on literature evidence. The literature evidence comes from the experience on HIL indexes by a single laboratory using Beckman instruments (6). fHb - cell-free hemoglobin. AST - aspartate aminotransferase. LD - lactate dehydrogenase. Cl - chloride. ALT - alanine aminotransferase. ALP - alkaline phosphatase. CRP - C-reactive protein.		

Free Hb results were entered in the spreadsheet where the algorithm was implemented and an automatic comment appeared indicating how to release the result. Results were alternately released as numeric, as “guide values”, or withheld with advice to contact the consultant.

Following the implementation of the algorithm on day nine of the hospital episode, there was a significant increase in the number of numeric and guide values that were released for this patient particularly analytes like creatinine and urea (Figure 1), enabling the hematology and nephrology teams to make more timely and appropriate decisions that impacted patient management. The percentage of results reported as numeric increased from 48% to 65%, the percentage released as guide values increased from 3% to 25%, while the proportion of requests with result withheld and a comment to contact the consultant is appended fell from 51% to 13%.

In particular, the introduction of the algorithm allowed dynamic changes in the patient’s kidney function to be monitored, allowing more confident decision-making on the initiation of continuous venovenous hemofiltration (day 12) and withdrawal of this intervention when her urine output improved, which was corroborated with an improvement in her biochemical parameters.

## Discussion

Hemolysis is a common occurrence in laboratory practice and is the most frequent reason for specimen rejection, as it influences the reliability of laboratory test results *via* biological and analytical mechanisms (*7*).

Hemolysis causes interference in the methods of analysis of biochemistry parameters by:

Increasing the concentration of the constituent by intracellular release, when the intracellular concentration is higher than extracellular (*e.g*., potassium, LD, aspartate aminotransferase (AST), magnesium or phosphate) (*8*, *9*).
Chemical interference. Some substances released by RBC interfere with chemical reactions. For example, fHb interferes in the measurement of bilirubin by disturbing the diazo-bilirubin reaction; oxyHb may lead to peroxide hydrogen (H_2_O_2_) that would result in peroxidation products of the diazo-bilirubin colour that would cause a decrease in the absorbance of the sample (*10*).
Spectrophotometric interference. The wavelength(s) at which the parameters are measured by spectrophotometry can overlap with the Hb absorption spectrum. In such cases, specific analytes may be over or under-estimated (*8*).
Interference by dilutional effect. The analytes whose concentration is lower inside the RBC compared with the extracellular fluid (*e.g*., sodium, chloride and glucose) (*9*).

Hemolysis indexes are established by manufacturers according to international and standardised protocols (CLSI EP07-A2) and should be based on comprehensive experimental studies using hemolyzed samples where lysis is produced by an overnight freeze-thaw cycle of whole blood samples (*11*). Hemolysis-free samples are spiked with this RBC lysate at different concentrations to achieve the calculation of specific cut-offs for hemolysis for each parameter. Manufacturers can establish the hemolysis cut-off quantitatively, so the index correlates to a specific concentration of fHb, or semi-quantitatively, where grades of HIs are on an ordinal scale and correlate to a range of fHb concentrations (*8*, *12*).

Handling samples and interpreting results in the context of hemolysis will vary depending on whether *in vitro* or *in vivo* hemolysis is the cause. *In vitro* hemolysis is the most common preanalytical error and can be identified visually. It can be avoided by repeating the blood sample collection and taking care with preanalytical factors (selection of venepuncture site and technique, tourniquet application and time, tube mixing and transport). *In vivo* hemolysis, in contrast, is much less common; less than 2% of all samples with detectable hemolysis are due to *in vivo* hemolysis (*13*). *In vivo* hemolysis is inherent to the patient´s clinical situation, difficult to visualise in low-grade cases and cannot be avoided. *In vivo* hemolysis should be considered differently from *in vitro* hemolysis, as the biochemistry results might be affected by the interference from hemolysis, but critical for clinical decision-making as they may reflect an accurate picture of the patient’s clinical state. In *in vivo* hemolysis, an increase in the concentration of high molecular weight RBC components (fHb and LD) may be observed in serum or plasma, while *in vitro* hemolysis, other components may be observed in the plasma or serum at an increased concentration (*e.g*., potassium, phosphate) (*8*). Rejection of suspected *in vivo* hemolysis samples may have critical consequences on the information available to clinicians to inform immediate clinical management. The elaboration of laboratory protocols, including further investigations with extra biochemistry or hematology testing in combination with clinical history, will typically resolve the distinction between *in vivo* and *in vitro* hemolysis, ensuring that critical results are analysed promptly for those with *in vivo* hemolysis (*14*).

Even where manufacturers suggest a cut-off to reject hemolyzed samples, these recommendations differ between platforms. There are no definitive guidelines describing the most suitable procedures for reporting and handling results of biochemical testing on hemolyzed specimens (*15*, *16*). One approach might be to quantify the fHb present in the sample to correlate with the hemolysis index and grade of interference as described in this case, however appropriate and full verification studies for analysis of fHb in serum or plasma by a method designed for measuring total Hb in whole blood should be developed (*17*). Some other clinical laboratories work with platforms that provide HI in a continuous scale that are convertible to exact Hb concentration and they would not need to measure the fHb by a hematology analyzer to follow the same procedure described for this case.

As illustrated in this case, even in the presence of interferants, reporting laboratory results can be essential for timely and appropriate patient management. Laboratories must be alert to the presence, type, grade, and effect of hemolysis to ensure accurate diagnostics and be alert to the limitations of automated HI systems.

Active communication between the laboratory and clinician is essential once the interference is recognised to help guide patient management. In this scenario, the hematology team would confirm that they suspect *in vivo* rather than *in vitro* hemolysis, and the blood bank laboratory can confirm this by performing DCT obtaining a positive result. Automatic blocking of results reporting in cases of *in vivo* hemolysis, as might be typical practice for *in vitro* hemolysis, can have detrimental consequences for the patient. Islam *et al*. described a confirmed case of true hyperkalemia in a patient diagnosed with hemolytic uremic syndrome and acute kidney failure where samples were grossly hemolyzed and automatically blocked by the analyzer. In that case, communication between the laboratory and clinical team did not occur, potassium results were not released according to the laboratory policy for hemolyzed samples, dialysis initiation was delayed, and the patient ultimately had a hyperkalemia-associated electromechanical dissociation cardiac arrest and died (*18*).

## What can be done to prevent such errors?

To prevent a delay in reporting results with potential interference from *in vivo* hemolysis, the following points should be considered:

Identify the kind of hemolysis present, whether it is a preanalytical or analytical interference and distinguish between *in vitro* or *in vivo* hemolysis. Check clinical details for underlying disease associated with *in vivo* hemolysis (*e.g*., hemolytic anemia), check other laboratory results or add extra testing to help make this distinction (*e.g*., haptoglobin, Hb, indirect bilirubin, reticulocytes, blood smear).
Ensure constant communication between the laboratory and clinical team in scenarios where validation of results is not automatic and where further investigation and interpretation may be needed.
Avoid *in vitro* hemolysis, which may confuse interpretation. Ensure all steps are taken to avoid preanalytical errors when collecting additional samples for a patient with *in vivo* hemolysis.
Since *in vivo* hemolysis cannot be avoided, specific procedures should be implemented to interpret and release reliable results promptly.
Measurement of fHb and correlation to a hemolysis grade interference may help to interpret results in patients with underlying hemolytic disease.
Comments should be added to all reports where parameters are potentially affected by the *in vivo* hemolysis. The laboratory should advise clinical teams on the risks and limitations of interpreting such results.

## Data Availability

The data generated during the current study are available from the corresponding author on reasonable request.
